# Tetra­aqua­(1,10-phenanthroline-5,6-dione-κ^2^ *N*,*N*′)cobalt(II) dinitrate

**DOI:** 10.1107/S1600536809017826

**Published:** 2009-05-20

**Authors:** Wen-Juan Shi

**Affiliations:** a Jiangxi Key Laboratory of Surface Engineering, Jiangxi Science and Technology Normal University, Jiangxi 330013, People’s Republic of China.

## Abstract

The asymmetric unit of the title compound, [Co(C_12_H_6_N_2_O_2_)(H_2_O)_4_](NO_3_)_2_, consists of a Co^II^ complex cation with twofold rotational symmetry and two nitrate anions. The Co^II^ atom has a distorted octa­hedral geometry with the basal plane occupied by two 1,10-phenanthroline-5,6-dione N atoms and two aqua O atoms, with the other two aqua ligands in axial positions. The aqua ligands are involved in extensive hydrogen bonding to nitrate and 1,10-phenanthroline-5,6-dione O atoms.

## Related literature

For related complexes of 1,10-phenanthroline-5,6-dione, see: Calderazzo *et al.* (1999 ▶); Fox *et al.* (1991 ▶); Onuegbu *et al.* (2007 ▶); Paw & Eisenberg (1997 ▶); Ruiz *et al.* (1999 ▶); Shavaleev *et al.* (2003 ▶). For the structures of the related phenanthroline and phendione derivatives of cobalt(II), see: Liu *et al.* (2008 ▶); Rubin-Preminger *et al.* (2008 ▶). For bond-length data, see: Allen *et al.* (1987 ▶).
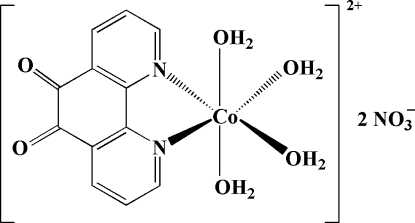


## Experimental

### 

#### Crystal data

[Co(C_12_H_6_N_2_O_2_)(H_2_O)_4_](NO_3_)_2_
*M* *_r_* = 465.20
Monoclinic, 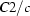

*a* = 12.7978 (12) Å
*b* = 10.6388 (10) Å
*c* = 13.0989 (12) Å
β = 105.248 (2)°
*V* = 1720.7 (3) Å^3^
*Z* = 4
Mo *K*α radiation
μ = 1.08 mm^−1^
*T* = 295 K
0.18 × 0.14 × 0.13 mm

#### Data collection

Bruker SMART APEX area-detector diffractometer
Absorption correction: multi-scan (*SADABS*; Sheldrick, 1996 ▶) *T*_min_ = 0.830, *T*_max_ = 0.873
4509 measured reflections
1695 independent reflections
1549 reflections with *I* > 2σ(*I*)
*R*_int_ = 0.020

#### Refinement

*R*[*F* ^2^ > 2σ(*F* ^2^)] = 0.051
*wR*(*F* ^2^) = 0.138
*S* = 1.04
1695 reflections
132 parameters
12 restraints
H-atom parameters constrained
Δρ_max_ = 0.68 e Å^−3^
Δρ_min_ = −0.61 e Å^−3^

### 

Data collection: *SMART* (Bruker, 2002 ▶); cell refinement: *SAINT* (Bruker, 2002 ▶); data reduction: *SAINT*; program(s) used to solve structure: *SHELXS97* (Sheldrick, 2008 ▶); program(s) used to refine structure: *SHELXL97* (Sheldrick, 2008 ▶); molecular graphics: *SHELXTL* (Sheldrick, 2008 ▶); software used to prepare material for publication: *SHELXTL*.

## Supplementary Material

Crystal structure: contains datablocks I, global. DOI: 10.1107/S1600536809017826/at2784sup1.cif

Structure factors: contains datablocks I. DOI: 10.1107/S1600536809017826/at2784Isup2.hkl

Additional supplementary materials:  crystallographic information; 3D view; checkCIF report

## Figures and Tables

**Table 1:** Hydrogen-bond geometry (Å, °)

*D*—H⋯*A*	*D*—H	H⋯*A*	*D*⋯*A*	*D*—H⋯*A*
O2*W*—H2*W*1⋯O4	0.85	2.21	2.834 (6)	130
O2*W*—H2*W*2⋯O2^i^	0.85	2.14	2.957 (6)	161
O1*W*—H1*W*1⋯O1^ii^	0.85	2.00	2.793 (5)	154
O1*W*—H1*W*2⋯O3^iii^	0.85	2.19	2.864 (6)	136

Symmetry codes: (i) 
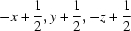
; (ii) 
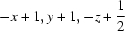
; (iii) 
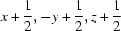
.

**Fig. 1.**
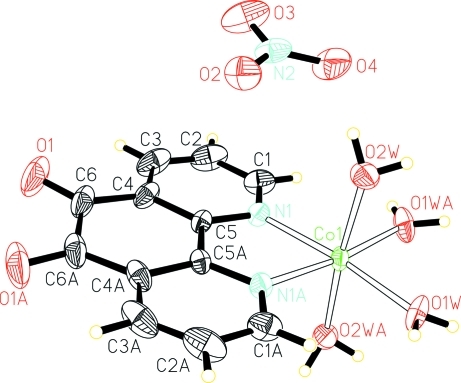
The molecular structure of the title compound, with displacement ellipsoids drawn at the 30% probability level. Hand H atoms are drawn as spheres of arbitrary radius. [Symmetry code, A: 1-x, y, 1/2-z].

**Fig. 2.**
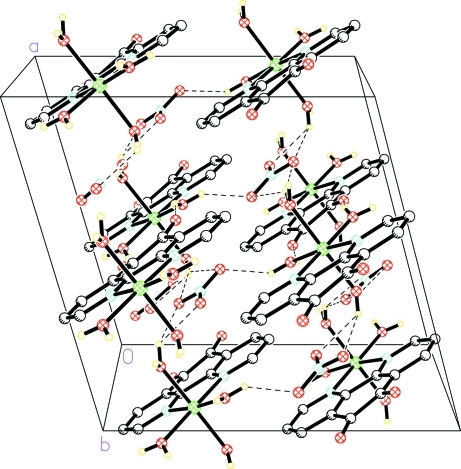
Packing diagram of the title structure, showing the intermolecular O—H···O hydrogen bonds as dashed lines.

## supplementary crystallographic information

### Comment

Phendione (1,10-phenanthroline-5,6-dione) is an excellent ligand that incorporates two functional groups with different coordination properties. Though phendione usually binds to metals through the imine N atoms (Onuegbu *et al.*, 2007), in some cases both the N and O donors are used simultaneously (Calderazzo *et al.*, 1999; Fox *et al.*, 1991; Paw & Eisenberg, 1997; Ruiz *et al.*, 1999; Shavaleev *et al.*, 2003). In this paper we report the synthesis and characterization of the title compound, [CoL(H_2_O)_4_].(NO_3_)_2_ (L = 1,10-phenanthroline-5,6-dione).

The molecular structure of the title compound, shown in Fig. 1, is made up of a [CoL(H_2_O)_4_]^2+^ cation and two nitrate anions, which the cations have twofold rotational symmetry. The cobalt atom is coordinated to the two N atoms of a phendione ligand and four aqua ligands to form distorted octahedral geometry. The C=O bond length in the phendione ligand [1.208 (6) Å] is comparable to those observed in other complexes of phendione (Allen *et al.*, 1987). The Co—N bond lengths [2.121 (3) Å] are similar to those values in related phenanthroline and phendione derivatives of cobalt(II) (Liu *et al.*, 2008; Rubin-Preminger *et al.*, 2008).

In addition to the strong O—H···O hydrogen bonds formed by the water ligands to both the nitrate and phendione O atoms, there are π-π stacking interactions between adjacent phendione ligands [perpendicular interplanar distance 3.582 (1) Å and centroid-to-centroid distance 3.823 (1) Å] (Fig. 2).

### Experimental

A solution of cobalt(II) nitrate hexahydrate (29.1 mg, 0.10 mmol) and phendione (21.0 mg, 0.10 mmol) in methanol (8 ml) was stirred for 2 h. After filtering, the filtrate was left at room temperature for about one week and purple, block-like crystals of the title compound appeared [yield: 18 mg (39%)].

### Refinement

The water H atoms were located in a difference Fourier map and refined with *U*_iso_(H) = 1.5*U*_eq_(O). The O—H distances of water were refined with idealized values of 0.85 Å. The aromatic H-atoms were positioned geometrically and refined using a riding model with d(C-H) = 0.93 Å, U_iso_=1.2U_eqeq_ (C).

### Crystal data

**Table:**

[Co(C_12_H_6_N_2_O_2_)(H_2_O)_4_](NO_3_)_2_	*F*(000) = 948
*M**_r_* = 465.20	*D*_x_ = 1.796 Mg m^−^^3^
Monoclinic, *C*2/*c*	Mo *K*α radiation, λ = 0.71073 Å
Hall symbol: -C 2yc	Cell parameters from 2754 reflections
*a* = 12.7978 (12) Å	θ = 2.5–26.8°
*b* = 10.6388 (10) Å	µ = 1.08 mm^−^^1^
*c* = 13.0989 (12) Å	*T* = 295 K
β = 105.248 (2)°	Block, purple
*V* = 1720.7 (3) Å^3^	0.18 × 0.14 × 0.13 mm
*Z* = 4	

### Data collection

**Table:**

Bruker SMART APEX area-detector diffractometer	1695 independent reflections
Radiation source: fine-focus sealed tube	1549 reflections with *I* > 2σ(*I*)
graphite	*R*_int_ = 0.020
φ and ω scans	θ_max_ = 26.0°, θ_min_ = 2.5°
Absorption correction: multi-scan (*SADABS*; Sheldrick, 1996)	*h* = −15→15
*T*_min_ = 0.830, *T*_max_ = 0.873	*k* = −5→13
4509 measured reflections	*l* = −16→15

### Refinement

**Table:**

Refinement on *F*^2^	Primary atom site location: structure-invariant direct methods
Least-squares matrix: full	Secondary atom site location: difference Fourier map
*R*[*F*^2^ > 2σ(*F*^2^)] = 0.051	Hydrogen site location: inferred from neighbouring sites
*wR*(*F*^2^) = 0.138	H-atom parameters constrained
*S* = 1.04	*w* = 1/[σ^2^(*F*_o_^2^) + (0.071*P*)^2^ + 5.1444*P*] where *P* = (*F*_o_^2^ + 2*F*_c_^2^)/3
1695 reflections	(Δ/σ)_max_ < 0.001
132 parameters	Δρ_max_ = 0.68 e Å^−^^3^
12 restraints	Δρ_min_ = −0.61 e Å^−^^3^

### Special details

**Table:**

Geometry. All esds (except the esd in the dihedral angle between two l.s. planes) are estimated using the full covariance matrix. The cell esds are taken into account individually in the estimation of esds in distances, angles and torsion angles; correlations between esds in cell parameters are only used when they are defined by crystal symmetry. An approximate (isotropic) treatment of cell esds is used for estimating esds involving l.s. planes.
Refinement. Refinement of F^2^ against ALL reflections. The weighted R-factor wR and goodness of fit S are based on F^2^, conventional R-factors R are based on F, with F set to zero for negative F^2^. The threshold expression of F^2^ > 2sigma(F^2^) is used only for calculating R-factors(gt) etc. and is not relevant to the choice of reflections for refinement. R-factors based on F^2^ are statistically about twice as large as those based on F, and R- factors based on ALL data will be even larger.

### Fractional atomic coordinates and isotropic or equivalent isotropic displacement parameters (Å^2^)

**Table:**

	*x*	*y*	*z*	*U*_iso_*/*U*_eq_	
Co1	0.5000	0.25979 (6)	0.2500	0.0379 (3)	
O1W	0.5719 (3)	0.3981 (3)	0.3584 (3)	0.0621 (10)	
H1W1	0.5858	0.4726	0.3425	0.075*	
H1W2	0.6036	0.3829	0.4228	0.075*	
O1	0.4265 (4)	−0.3399 (4)	0.1544 (5)	0.1088 (17)	
O2W	0.3649 (3)	0.2686 (3)	0.3112 (3)	0.0554 (9)	
H2W1	0.3018	0.2370	0.2998	0.066*	
H2W2	0.3376	0.3407	0.3149	0.066*	
O2	0.2233 (3)	0.0263 (4)	0.2267 (3)	0.0784 (12)	
O3	0.1111 (4)	0.0285 (6)	0.0756 (4)	0.107 (2)	
O4	0.1792 (4)	0.2017 (5)	0.1484 (4)	0.0996 (18)	
N1	0.4417 (3)	0.1042 (3)	0.1501 (3)	0.0394 (8)	
N2	0.1708 (3)	0.0851 (5)	0.1490 (3)	0.0649 (13)	
C1	0.3828 (4)	0.1087 (6)	0.0491 (4)	0.0585 (12)	
H1	0.3646	0.1869	0.0176	0.070*	
C2	0.3481 (5)	0.0028 (7)	−0.0099 (4)	0.0761 (15)	
H2	0.3086	0.0097	−0.0802	0.091*	
C3	0.3716 (5)	−0.1113 (6)	0.0347 (5)	0.0735 (14)	
H3	0.3470	−0.1836	−0.0040	0.088*	
C4	0.4336 (4)	−0.1200 (4)	0.1406 (4)	0.0515 (11)	
C5	0.4673 (3)	−0.0087 (4)	0.1946 (3)	0.0353 (8)	
C6	0.4594 (3)	−0.2412 (4)	0.1964 (4)	0.0679 (14)	

### Atomic displacement parameters (Å^2^)

**Table:**

	*U*^11^	*U*^22^	*U*^33^	*U*^12^	*U*^13^	*U*^23^
Co1	0.0443 (5)	0.0219 (4)	0.0489 (5)	0.000	0.0149 (4)	0.000
O1W	0.094 (3)	0.0341 (16)	0.065 (2)	−0.0210 (17)	0.0333 (19)	−0.0165 (15)
O1	0.133 (4)	0.047 (2)	0.169 (4)	−0.030 (2)	0.080 (3)	−0.043 (2)
O2W	0.0501 (18)	0.0530 (19)	0.069 (2)	0.0035 (14)	0.0271 (17)	0.0139 (16)
O2	0.075 (3)	0.090 (3)	0.065 (2)	0.006 (2)	0.010 (2)	0.033 (2)
O3	0.092 (3)	0.166 (6)	0.058 (3)	−0.050 (3)	0.011 (2)	0.001 (3)
O4	0.079 (3)	0.089 (3)	0.125 (4)	−0.014 (3)	0.016 (3)	0.063 (3)
N1	0.0454 (18)	0.0375 (18)	0.0322 (16)	−0.0019 (15)	0.0046 (14)	0.0049 (14)
N2	0.048 (2)	0.092 (4)	0.054 (2)	−0.014 (2)	0.0108 (19)	0.027 (2)
C1	0.052 (2)	0.079 (3)	0.039 (2)	−0.004 (2)	0.0033 (18)	0.010 (2)
C2	0.066 (3)	0.117 (4)	0.042 (2)	−0.025 (3)	0.007 (2)	−0.015 (2)
C3	0.074 (3)	0.090 (3)	0.062 (3)	−0.036 (3)	0.028 (2)	−0.041 (2)
C4	0.060 (3)	0.047 (2)	0.057 (2)	−0.0194 (19)	0.0323 (19)	−0.0230 (18)
C5	0.0425 (19)	0.0318 (18)	0.0342 (19)	−0.0051 (15)	0.0143 (16)	−0.0032 (14)
C6	0.083 (3)	0.036 (2)	0.104 (4)	−0.012 (2)	0.059 (3)	−0.0202 (19)

### Geometric parameters (Å, °)

**Table:**

Co1—O1W	2.084 (3)	O4—N2	1.245 (7)
Co1—O1W^i^	2.084 (3)	N1—C5	1.338 (5)
Co1—O2W	2.091 (3)	N1—C1	1.340 (5)
Co1—O2W^i^	2.091 (3)	C1—C2	1.372 (9)
Co1—N1	2.121 (3)	C1—H1	0.9300
Co1—N1^i^	2.121 (3)	C2—C3	1.346 (10)
O1W—H1W1	0.8500	C2—H2	0.9300
O1W—H1W2	0.8502	C3—C4	1.408 (8)
O1—C6	1.208 (6)	C3—H3	0.9300
O2W—H2W1	0.8501	C4—C5	1.388 (6)
O2W—H2W2	0.8500	C4—C6	1.476 (7)
O2—N2	1.232 (6)	C5—C5^i^	1.473 (8)
O3—N2	1.218 (6)	C6—C6^i^	1.511 (10)
			
O1W—Co1—O1W^i^	90.1 (2)	C1—N1—Co1	126.6 (3)
O1W—Co1—O2W	88.21 (14)	O3—N2—O2	119.6 (6)
O1W^i^—Co1—O2W	88.18 (14)	O3—N2—O4	121.7 (5)
O1W—Co1—O2W^i^	88.18 (14)	O2—N2—O4	118.7 (5)
O1W^i^—Co1—O2W^i^	88.21 (14)	N1—C1—C2	122.7 (5)
O2W—Co1—O2W^i^	174.89 (19)	N1—C1—H1	118.7
O1W—Co1—N1	173.05 (14)	C2—C1—H1	118.7
O1W^i^—Co1—N1	96.32 (15)	C3—C2—C1	119.6 (5)
O2W—Co1—N1	94.55 (14)	C3—C2—H2	120.2
O2W^i^—Co1—N1	89.44 (13)	C1—C2—H2	120.2
O1W—Co1—N1^i^	96.32 (15)	C2—C3—C4	119.4 (5)
O1W^i^—Co1—N1^i^	173.05 (14)	C2—C3—H3	120.3
O2W—Co1—N1^i^	89.44 (13)	C4—C3—H3	120.3
O2W^i^—Co1—N1^i^	94.55 (14)	C5—C4—C3	117.7 (5)
N1—Co1—N1^i^	77.36 (18)	C5—C4—C6	119.6 (4)
Co1—O1W—H1W1	125.1	C3—C4—C6	122.7 (4)
Co1—O1W—H1W2	123.5	N1—C5—C4	122.4 (4)
H1W1—O1W—H1W2	110.1	N1—C5—C5^i^	116.1 (2)
Co1—O2W—H2W1	139.2	C4—C5—C5^i^	121.5 (3)
Co1—O2W—H2W2	117.1	O1—C6—C4	121.8 (5)
H2W1—O2W—H2W2	89.0	O1—C6—C6^i^	119.7 (4)
C5—N1—C1	118.2 (4)	C4—C6—C6^i^	118.0 (3)
C5—N1—Co1	115.2 (2)		
			
O1W^i^—Co1—N1—C5	−176.8 (3)	C2—C3—C4—C6	177.7 (5)
O2W—Co1—N1—C5	−88.2 (3)	C1—N1—C5—C4	−1.0 (6)
O2W^i^—Co1—N1—C5	95.0 (3)	Co1—N1—C5—C4	178.8 (3)
N1^i^—Co1—N1—C5	0.2 (2)	C1—N1—C5—C5^i^	179.6 (4)
O1W^i^—Co1—N1—C1	2.9 (4)	Co1—N1—C5—C5^i^	−0.6 (5)
O2W—Co1—N1—C1	91.6 (4)	C3—C4—C5—N1	0.8 (6)
O2W^i^—Co1—N1—C1	−85.2 (4)	C6—C4—C5—N1	−176.5 (4)
N1^i^—Co1—N1—C1	180.0 (5)	C3—C4—C5—C5^i^	−179.8 (5)
C5—N1—C1—C2	−0.1 (7)	C6—C4—C5—C5^i^	2.9 (7)
Co1—N1—C1—C2	−179.8 (4)	C5—C4—C6—O1	175.7 (3)
N1—C1—C2—C3	1.3 (9)	C3—C4—C6—O1	−1.4 (6)
C1—C2—C3—C4	−1.5 (9)	C5—C4—C6—C6^i^	−12.2 (6)
C2—C3—C4—C5	0.5 (8)	C3—C4—C6—C6^i^	170.7 (4)

Symmetry codes: (i) −*x*+1, *y*, −*z*+1/2.

### Hydrogen-bond geometry (Å, °)

**Table:**

*D*—H···*A*	*D*—H	H···*A*	*D*···*A*	*D*—H···*A*
O2W—H2W1···O4	0.85	2.21	2.834 (6)	130
O2W—H2W2···O2^ii^	0.85	2.14	2.957 (6)	161
O1W—H1W1···O1^iii^	0.85	2.00	2.793 (5)	154
O1W—H1W2···O3^iv^	0.85	2.19	2.864 (6)	136

Symmetry codes: (ii) −*x*+1/2, *y*+1/2, −*z*+1/2; (iii) −*x*+1, *y*+1, −*z*+1/2; (iv) *x*+1/2, −*y*+1/2, *z*+1/2.
